# Ganglioneuroma of the sphenoid wing: a case report and literature review 

**DOI:** 10.5414/NP300376

**Published:** 2011-10-18

**Authors:** E. Aktüre, M.S. Salamat, H. Korkmaz, M.K. Başkaya

**Affiliations:** 1 Department of Neurological Surgery; 2 Department of Pathology and Laboratory Medicine, School of Medicine and Public Health, University of Wisconsin, Madison, WI, USA

**Keywords:** ganglioneuroma, neural crest derived tumor, sphenoid wing, neuroblastoma, calvarial tumor

## Abstract

Ganglioneuromas (GNs) are well-differentiated, slow-growing, benign tumors that are quite rare and usually found in the posterior mediastinum and retroperitoneum. They are composed of ganglion and Schwann cells and their origin remains in dispute. GNs have been reported as intraosseous lesions, such as in temporal and orbital bones. There are rare reports of intracranial lesions, mostly in the pituitary fossa. Most GN patients are children and are clinically asymptomatic. Diagnosis of GN requires histopathologic evaluation since no specific clinical or radiologic diagnostic features have been identified. We report the case of a 35-year-old man with recurrent sinusitis whose radiologic workup revealed a lytic right sphenoid wing lesion with microcalcifications. He underwent gross-total resection of the lesion and the pathologic findings were diagnostic of ganglioneuroma. To the best of our knowledge, this is the first reported case of sphenoid wing GN. The nature and origin of this tumor are discussed, and the GN literature is reviewed.

## Introduction 

Ganglioneuromas (GNs) are benign tumors of the peripheral nervous system that are seldom seen in the cranial bones or intracranially. They are composed of mature ganglion cells, occasional satellite or “capsular” cells, unmyelinated axons and Schwann cells [1]. While the origin of GNs remains unclear, such tumors have been observed to arise from maturation of neuroblastomas either spontaneously or particularly, after chemoradiotherapy [2, 3]. However, the distribution differences between neuroblastomas and GNs indicate that de novo development of these tumors exists and is more common. When compared to other benign tumors of the peripheral nervous system such as schwannomas and neurofibromas, GNs are quite rare [4]. 

Whether arising in the soft tissue or bone, GNs can increase in size and cause compression of adjacent tissue [5]. Intraosseous locations include long bones, vertebrae and cranium [5]. Cranial locations include the orbit and the temporal bones [6, 7, 8]. Vertebral locations include the sacrum and craniocervical junction. Reported intracranial cases are in the older literature and mostly intrasellar [9, 10]. Based on descriptions given in the older literature, many of these intracranial tumors are more consistent with gangliogliomas, hamartomas and metaplastic neurons in pituitary adenomas. 

Additionally, familial disposition as well as an association with neurofibromatosis, Turner Syndrome and multiple endocrine neoplasia syndrome Type-B has been reported [11]. Rarely malignant transformation has been reported either spontaneously or after radiotherapy [12, 13, 14]. Some rare cases of GN are found to secrete sufficient quantities of vasoactive intestinal polypeptide (VIP), vanilmandelic acid (VMA) and homovanilic acid (HVA) to cause flushing, diarrhea and other symptoms of catecholamine excess [15]. 

These slow growing benign tumors are not always symptomatic but if symptomatic, it is usually due to compression of autonomic or somatic peripheral nerves. In this paper, we present a right sphenoid wing GN that was found incidentally in a 35-year-old man. 

## Clinical summary 

A 35-year-old man who had recently undergone septoplasty and bilateral turbinate reduction for frequent episodes of sinusitis was found to have a lytic lesion with smooth borders in the right sphenoid wing on preoperative computerized tomography (CT) scan. After surgery, he was referred for neurosurgical evaluation of the asymptomatic mass. No abnormality was found on the general or neurological examination. Cranial nerve examination in particular showed that facial sensation was intact. 

## Radiology 

**Figure 1.**
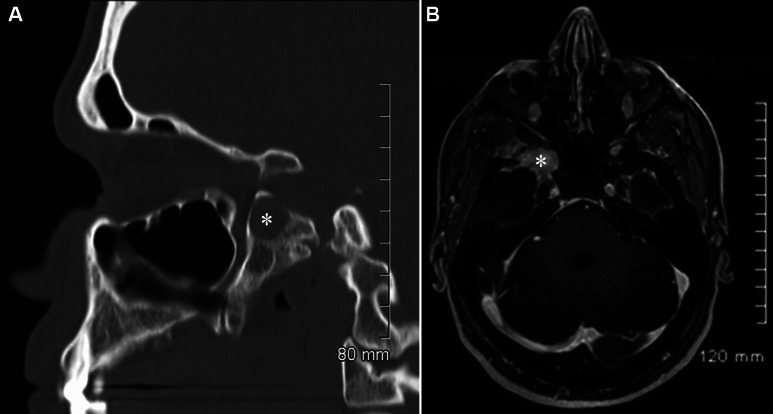
A: Sagittal reformats of the axial non-contrast CT scan revealing the well-demarcated, oval lesion (asterisk) at the sphenoid bone. B: Post-contrast fat saturated axial T1-weight cranial MRI revealing enhancing well-demarcated lesion (asterisk) within the right sphenoid wing around the foramen rotundum.

CT scan of the head revealed a fibro-osseous, well-demarcated lesion in the right sphenoid wing with remodeling and sclerosis of the adjacent bone (Figure 1A). Magnetic resonance imaging (MRI) also revealed a right sphenoid wing lesion measuring approximately 3 cm in the long axis extending inferiorly to pterygoid plate and superiorly to the sphenoid wing with some bone remodeling (Figure 1B). It was low in signal intensity on non-contrast T1-weighted images, heterogeneously isointense on T2-weighted sequences and demonstrated prominent enhancement. The right foramen rotundum appeared involved by the lesion. Differential diagnosis included intraosseous meningioma, metastatic tumors and granulomatous infection. 

## Surgery 

Due to uncertainty regarding the nature of this lesion, the patient elected for excisional biopsy. He underwent a right cranio-orbital approach for microsurgical gross total resection of the lesion. Extradural dissection was performed and the dura was reflected from the greater wing of the sphenoid bone to expose the lesion. The V2 and V3 branches of the trigeminal nerve were identified, and the lesion was found near the foramen rotundum. The trigeminal ganglion and/or trigeminal recess (Meckel’s cave) was not exposed since the lesion was not reaching posteriorly. The V2 branch of the trigeminal nerve was severely compressed in the foramen by the tumor. However, no nerve infiltration was observed. Nerve decompression was achieved by microsurgical resection of the tumor. 

## Pathological findings 

**Figure 2.**
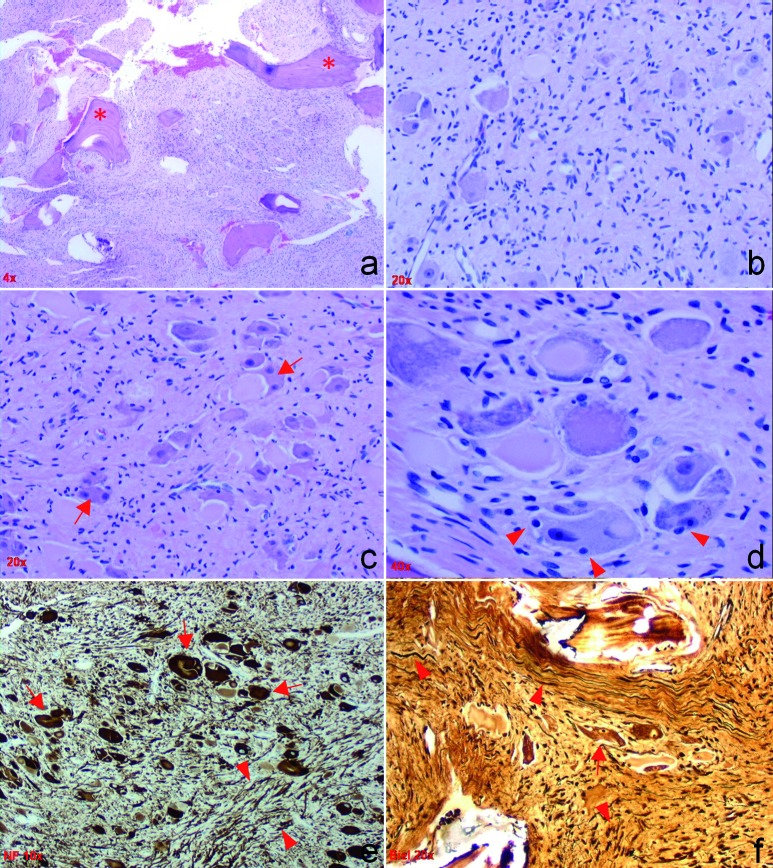
a: H&E stain at low (4 ×) magnification reveals an intraosseous tumor of moderate cellularity and boney specules, marked by asterisk. b: At higher magnification (20 x), randomly distributed large and dysplastic ganglion cells are seen in a background of neoplastic spindle cells with scant cytoplasm. c, d: H & E stains reveal irregular clusters of dysplastic ganglion cells with abnormal distribution of the chromophylic substance, binucleated cells (arrows) and infrequent satellite cells (arrowheads). e, f: At 10 × magnification, the neurofilament (directed against 70 kDa and 200 kDa polypeptides of non-phosphorylated neurofilament) (e) and at 20 × magnification, sections stained with silver (f) reveal bizarre and haphazardly oriented axons (arrowheads) as well as few large ganglion cells (arrows).

Histologically, the excised tissue revealed a well-differentiated intraosseous neoplasm, replacing the bone marrow and consisting of a large number of mature ganglion cells in a moderately cellular stroma of spindle cells (Figure 2a – f). The ganglion cells were large and in clusters, or randomly scattered, containing variable chromophylic/Nissl substance and rare cytoplasmic vacuoles. They revealed mildly pleomorphic, vescicular nuclei, occasional binucleation and paucity of satellite cells. Neuroblastic cells and immature neurons were absent in the lesion. The tumor revealed abundant elongated axons with Bielschowsky silver and lacked myelin with Luxol fast blue stain. The ganglion cells were positively immunolabeled with synaptophysin antibody and their processes stained with silver. The neurofillament antibody immunolabeled both cell bodies and processes of the ganglion cells. Normal ganglionic or peripheral nerve elements were not seen. Postsurgical neurological examination did not reveal any deficits. CT studies performed 2 months after surgery did not reveal residual or recurrent tumor. 

## Discussion 

Ganglioneuroma (GN) is classified as a benign well-differentiated neoplasm of the peripheral nervous system (PNS) [2]. GNs are composed of mature ganglion and Schwann cells, and accurate diagnosis of GN without tissue examination is difficult [16]. Most often GNs are asymptomatic, incidental masses discovered on a routine radiographic study [17]. They become symptomatic when they compress and/or displace adjacent structures. The first documented case of GN was reported by Loretz in 1870 [18]. GNs are classically found within the posterior mediastinum (41.5%), pelvis and retroperitoneum (the suprarenal medulla is a favorite site) [1, 2, 3, 4, 11, 13, 16, 19, 20, 21]. These sites are usually associated with autonomic ganglia, especially sympathetic ganglia [1]. In our case during surgery, this intraosseous lesion was not noted to be contiguous with the pterygopalatine fossa, pterygopalatine ganglion or trigeminal (Gasserian) ganglion. Microscopy also did not reveal any peripheral nerve or ganglion. Rarely, GNs have been observed in the spinal cord, cranial nerve ganglia, mandible, tongue, parapharyngeal tissue, gastrointestinal tract, bladder, visceral ganglia, uterus, ovary, spermatic cord, testes, prostate, skin, and bone [5, 13, 22, 23]. Involvement of the central nervous system (CNS) has been described but is extremely rare [3]. Keefe reported a case of left posterior cranial fossa GN and mentioned that 11 cerebellar cases had been reported up to that point [24]. Ozluoglu et al. reported a lesion in the internal auditory canal that presented with hearing loss [25]. In fact, based on available microscopic descriptions, most of the CNS lesions listed in the older literature very likely represent intracranial hamartomas or gangliogliomas, which are both lesions of the CNS and not the PNS. Lesions associated with the cranial vault have been reported in the temporal bone [26] and the orbital bone [7]. Also, there are case reports of lesions in the orbit [6, 7], optic nerve and chiasm [27]. Our case is the first lesion reported in the greater sphenoid wing. 

GNs are usually seen before the second decade of life and rarely after the sixth [4]. The median age at diagnosis has been reported to be approximately 7 years (5.5 – 10 years) [17]. There are different reports regarding gender predilection. Some studies reveal no gender predilection whereas some mention slight female predominance [11, 19, 21]. Our case is further unique in that the GN is identified in a 35-year-old man without a prior history of neuroblastoma or association with a familial disorder, thus representing a case of sporadic benign neoplasm arising de novo in an unusual location. 

Considering the radiological findings, age bracket, and the location of the lesion, our preoperative diagnosis favored intraosseous meningioma. However, microscopic tissue examination reveals typical features of a ganglioneuroma. GNs lack cellular atypia, mitotic activity, and necrosis [28], and overall have relatively better prognosis than most of the other tumors derived from the neural crest. Malignant transformation is very rare and total resection is the most important treatment modality. Radiation and chemotherapy have no role in the treatment of these tumors [16, 17, 29]. 

In summary, cranial ganglioneuromas are extremely rare. Being benign tumors, gross-total surgical excision is considered to be curative. However, clinical follow up of the patient is recommended since rare cases of malignant transformation have been reported. In this case report, we present the first case of ganglioneuroma involving the sphenoid wing. 

## Acknowledgments 

The authors thank Traci Niesen HT (ASCP), Sally A. Drew MT (ASCP), and Linda A. Sebree HT (ASCP) for their excellent technical assistance, as well as Korise Rasmusson PhD and Gregory Kujoth, PhD, for their assistance with preparation of this report.
